# Milk Sickness

**Published:** 1840-01

**Authors:** 


### MILK SICKNESS, *alias* SICK STOMACH.

This endemic of the West, to which science has not yet given a name, and even sometimes professes to doubt the existence, continues to attract the attention of the people and country practitioners, in various parts of Ohio, Indiana, Illinois, Kentucky and Tennessee. Sometime since, we received two communications concerning it. One from Miami county, the other, accompanied with specimens of a plant supposed to be the remote cause, from Fayette county, in the State of Ohio. The former by an intelligent but non-professional gentleman, presents as the poisonous plant, supposed to produce the disease, the *Rhus radicans*; but as this vine grows universally in the West, while the malady in question, is limited to particular spots, we cannot concur in the hypothesis. The plant transmitted to us by the latter, is the *Eupatorium ageratoides*, found *every where*, in our fertile woods; and, therefore, not likely to be the cause, of a disease perseveringly limited to certain localities. Moreover, judging it by the taste, it is quite inert; and indeed the whole genus to which it belongs, except the *perfoliatum*, appear to be destitute of active qualities, and that species, is by no means of a noxious character.

In connexion with this subject we record the following fact. In the month of July last, about twenty of the boarders, in the Hotel of Mr. Madeira, Chillicothe, Ohio, were attacked, in one, two, or three hours after breakfast, with nausea and vomiting. In some, the latter was violent, and accompanied with spasms of the stomach, and a degree of prostration, from which they did not entirely recover for three or four days. Of course, this affection was ascribed to something eaten at the table, but the only article taken by the whole, was *butter*; and that butter, it was ascertained had been brought from an adjoining county in which the milk sickness prevails. Many facts of this kind have been reported by the people in different parts of the West, but, generally, discredited by the profession. We beg leave to commend the whole subject to our country friends, and shall be happy to give publicity to their observations and experiments. D.

